# Coronary Artery Aneurysms: A Review of the Epidemiology, Pathophysiology, Diagnosis, and Treatment

**DOI:** 10.3389/fcvm.2017.00024

**Published:** 2017-05-05

**Authors:** Sara Abou Sherif, Ozge Ozden Tok, Özgür Taşköylü, Omer Goktekin, Ismail Dogu Kilic

**Affiliations:** ^1^Cardiovascular Research Division, Kings College London, London, UK; ^2^Department of Cardiology, Memorial Hospital, Istanbul, Turkey; ^3^Ozel Saglik Hospital, Denizli, Turkey; ^4^Department of Cardiology, Pamukkale University, Denizli, Turkey

**Keywords:** coronary artery aneursym, atherosclerosis, coronary artery disease, coronary stent, coronary artery ectasia, coronary aneurysm

## Abstract

Coronary artery aneurysms (CAAs) are uncommon and describe a localized dilatation of a coronary artery segment more than 1.5-fold compared with adjacent normal segments. The incidence of CAAs varies from 0.3 to 5.3%. Ever since the dawn of the interventional era, CAAs have been increasingly diagnosed on coronary angiography. Causative factors include atherosclerosis, Takayasu arteritis, congenital disorders, Kawasaki disease (KD), and percutaneous coronary intervention. The natural history of CAAs remains unclear; however, several recent studies have postulated the underlying molecular mechanisms of CAAs, and genome-wide association studies have revealed several genetic predispositions to CAA. Controversies persist regarding the management of CAAs, and emerging findings support the importance of an early diagnosis in patients predisposed to CAAs, such as in children with KD. This review aims to summarize the present knowledge of CAAs and collate the recent advances regarding the epidemiology, etiology, pathophysiology, diagnosis, and treatment of this disease.

## Introduction

Coronary artery aneurysms (CAAs) are uncommon and describe a localized dilatation of a coronary artery segment more than 1.5-fold the normal size of adjacent normal segments (1). Several large angiographic series have contributed to the understanding of CAAs. This review aims to summarize the present knowledge on CAAs.

## Definition and Classification of CAAs

Coronary artery aneurysms describe local dilatations in the coronary artery that are 1.5-fold greater than normal adjacent segments. Coronary artery ectasias, on the other hand, appear as diffuse arterial dilatations, where the length of the dilated segment is found to be more than 50% of the diameter (1). Depending on the integrity of the vessel wall, aneurysms can be classified as true or false aneurysms (pseudoaneurysms). Pseudoaneurysms are dilatations of the vessel, with single or double layers of the vessel wall (rather than the normal three-layered structure), because of disruption of the media and external elastic membrane. They are usually caused by blunt chest trauma or coronary intervention. In contrast, a true arterial aneurysm is a fusiform or saccular dilatation of the artery, involving the three layers of the vessel’s tunica. Some authors describe dilatations that exceed 4 cm in diameter as giant coronary aneurysms (2).

## Epidemiology

The overall incidence of CAAs ranges from 0.3 to 5.3%; a pooled analysis states a mean incidence of 1.65% (1–8). The incidence of giant CAAs is as low as 0.02%, while the incidence of those associated with congenital artery fistulae are 5.9% (3). Nevertheless, the true burden of CAAs and ectasia may be currently underestimated. The use of non-invasive technology such as computed tomography (CT) and magnetic resonance (MR) coronary angiography may increase the recognition of this entity (5). A wide variation exists in the occurrence of CAAs, which could mean that the reported incidences may not accurately represent the wider population. This could be explained by many factors such as the varied angiographic criteria used to define CAAs (2–5) or the differing definitions of CAAs used by various institutions (9). For example, some studies include both aneurysmal coronary disease and ectasia in their CAA incidence calculations, while others do not (9, 10). Moreover, each angiographic diagnosis of a CAA is operator dependent; therefore, any interobserver variability may contribute to the differences in the incidence data reported (9). Likewise, the geographical variation in these studies highlights important genetic and environmental influences that affect the incidence of CAAs, of which the former shall be discussed later. The CAA incidence has been shown to be lower in Asia than in North America and Europe. In a study on 302 patients with Kawasaki disease (KD), the incidence of CAAs in patients of Asian, Caucasian, and African ethnicity was 10.3, 6.9, and 1.2%, respectively (10). In addition to that, a study carried out in India reported a large 10–12% incidence of CAA, further highlighting the possible genetic and/or environmental susceptibility to CAAs (11).

The incidence of CAA is higher in men than in women, 2.2 versus 0.5%, respectively (6, 12). Results from 5,000 angiograms attained from a single institution in the United Kingdom showed that 70 patients found to have coronary ectasia and 54 had significant atherosclerotic coronary artery disease (ACAD), and there was a significant gender difference in the incidence of coronary dilatations, with 0.5% of females and 2.2% of males with ACAD having concomitant coronary artery ectasia (3). Furthermore, although CAAs can be seen at any age, those related to atherosclerosis usually appear later in life compared to congenital CAAs or those with an underlying inflammatory nature (13).

### Anatomical Distribution

The right coronary artery is the most commonly affected, and it is involved in 40–70% of CAAs. The left circumflex (23.4%) or the left anterior descending artery (32.3%) follows depending on the study (14–18). Involvement of three coronary vessels or the left main is a much rarer occurrence (3.5%) (4, 19). Atherosclerotic or inflammatory coronary aneurysms are usually multiple and involve more than one coronary artery. In contrast, congenital, traumatic, or dissecting aneurysms typically involve a single artery (20).

## Pathogenesis and Etiology

The pathophysiological mechanisms behind CAAs remain controversial. While the underlying pathological processes vary with each etiology, they appear to contribute to a unifying mechanism of inherent vessel wall weakening and subsequent dilatation (21).

### CAA and Coronary Artery Disease

The most common cause of CAAs is atherosclerosis, which has been linked to 50% of CAAs diagnosed in adults (22). In a large angiographic series, the Coronary Artery Surgery Study (CASS) registry (5, 23), investigators reported a prevalence of 4.9% (24), which exceeded the rates of CAAs in many other angiographic studies (0.37–2.53%) (3). As a part of the CASS registry, successive angiograms from over 20,000 patients were performed and analyzed (12). When 1,000 patients with aneurysms and concomitant coronary artery disease were compared with patients with coronary artery stenoses alone (no aneurysms), they were more likely to be male, to have suffered a myocardial infarction in the past, and to have a three-vessel atherosclerotic disease. Closer analysis of 978 patients with CAAs revealed that 957 of them had concomitant coronary artery stenosis. Similar results were observed in a retrospective study from Greece, where 5.3% (203) of 3,900 patient angiograms analyzed were found to have coronary aneurysmal disease; of these 203 patients, 173 had associated significant ACAD (25). Both these studies revealed no significant difference in the incidence of risk factors such as hyperlipidemia, hypertension, diabetes, peripheral vascular disease, myocardial infarction, smoking, or the presence of a family history of CAD. While the increased association between CAAs and coronary artery disease could be predictable and unsurprising across a population who are more likely to receive a diagnostic angiogram, the histopathological features of coronary aneurysms further support the pathophysiological link between these two diseases. Histological examination of atherosclerotic CAAs revealed hyalinization and lipid deposition leading to disturbance of the intimal and medial layers of the vessel wall and destruction of the muscular elastic components (20, 26–31). Further reports highlighted the presence of focal areas of calcification and fibrosis as well as large cholesterol crystals. Such features weaken the vessel wall and decrease its elasticity, ultimately reducing the vessel’s tolerance to intraluminal pressures of blood flow, thus predisposing it to subsequent dilatation and aneurysm formation. Long-term transmural inflammation, which is characteristic of atherosclerotic disease, further contributes to this process of vessel wall weakening (26–32). Additional literature speculates that the chronic overstimulation of the vasodilator nitric oxide and the local mechanical stresses from stenoses may contribute to the weakening of the medial wall of the coronary artery (19).

### Genetic Contribution in ACAD-Associated CAAs

In support of the predisposition of CAAs in patients with coronary artery disease, several genome-wide association studies (GWAS) have discovered the association of variants on chromosome 9p21.3 with CAD, and this locus was also associated with an increased risk of various other diseases including abdominal aortic and intracranial aneurysms. The 9p21.3 risk allele has been linked to an altered proliferative phenotype that promotes adverse vascular remodeling (33–35). Given the similar pathogenesis of CAAs with that of larger vessel, further studies are essential to quantify the extent of this potential overlap in the genetic predispositions to both atherosclerotic and aneurysmal disease. Moreover, further investigations to clarify the extent to which genetic polymorphisms contribute to the underlying pathophysiology of CAAs are essential in addressing whether the identification of patients with CAD at a significant risk of concomitant CAA will be possible. While the impact of CAAs on patient’s survival with coronary artery disease remains under debate, CAA sufferers generally carry a worse prognosis than the general population (4, 22), thus methods of risk stratification may add valuable clinical information.

### CAA and Percutaneous Coronary Interventions

Ever since the dawn of the interventional era, there has been a growing concern about the occurrence of CAAs following the treatment of ACAD with PCI, including balloon angioplasty, stent implantation (36–39), and in particularly, the use of drug-eluting stents (DESs) (8, 40, 41).

### CAA and Stent Implantation

Ahn et al. assessed the occurrence of DES-associated CAAs in 3,616 consecutive patients (a total of 4,419 lesions) who underwent follow-up angiography after DES implantation. Thirty-four CAAs (0.76% per lesion) in 29 patients (0.8% per patient) were detected at follow-up at a mean number of 414 ± 213 days following DES implantation (40). Present literature demonstrates that the incidence of CAAs associated with stent implantation ranges from 1.25 to 3.9% (8, 36); however, this is not representative of the true occurrence due to the lack of overall data and the specific time in which CAAs develop after DES implantation, which is affected by various parameters such as the time of angiographic investigation following stent implantation as well as the varying definitions of CAA used and different inclusion criteria for further investigations after PCIs. Several mechanisms have been proposed to explain the formation of stent-related CAAs, including the formation of a coronary dissection and last stent malapposition (42). The use of oversized, high-pressure balloons during PCI can cause dissection of the vessel. It has also been shown that the development of CAAs is related to the use of bailout stenting after a coronary dissection due to PTCA (43).

While the mechanical factors, mentioned above, drive the formation of aneurysms following bare metal stents, DESs carry additional features that contribute to the weakening of a coronary vessel (44). First, DESs are primed with various cytotoxic antirestenosis drugs, which are known to suppress smooth muscle and endothelial cell proliferation, thus preventing restenosis (44–46). However, this antiproliferative nature of DES has been shown to increase the risk of CAAs *via* mechanisms of delayed neointimal healing and reendotheliazation (44, 45, 47). Studies examining the actions of drugs in DESs have demonstrated reduced neointimal formation, accompanied with persistent fibrin deposition, macrophage infiltration, and an overall decrease in the number of smooth muscle cells at both 28 and 180 days following the implantation of DES in rabbit iliac and porcine coronary arteries (44, 48–52). Second, a significant vascular inflammatory response to DES resulting in increased eosinophilic/heterophilic infiltration into the vessel wall has been observed (53–55). The likely explanation for these findings describes a local hypersensitivity reaction to the polymer carrying the drug in the DES. Overall, hypersensitivity reactions and local cytotoxic effects of drugs contribute to the disruption and weakening of all three layers of the arterial wall, leading to their expansion and predisposing the vessel to aneurysmal disease (44).

In their study, Alfonso et al. analyzed 1,197 patients whom had undergone late angiographic evaluation after DES implantation. CAAs were found to have developed in 15 patients (1.25%, 95% CI: 0.58–1.93); the average time from DES implantation to CAA formation was found to be 313 ± 194 days. The 1-year event-free survival was 49 ± 14% and was found to be related to the size of the CAA. The clinical presentation of the CAAs varied from an acute myocardial infarction (in two patients), unstable angina (in four patients) to remaining clinically silent in nine patients. This study showed that, although DES-related aneurysms are rare, they can carry a potentially fatal outcome for patients; one of the patients who was asymptomatic on CAA diagnosis died a year later due to a CAA-related myocardial infarction (8). Additional studies are required to determine the implications of DES-associated CAAs and raise this as a potentially rare but fatal complication among interventionists. Larger cohort studies are needed to reflect the actual burden of CAAs as a complication of DES and further the understanding of the long-term safety of DES.

Bioabsorbable vascular scaffolds (BVS) are also emerging materials in PCI (56–63). Various processes can drive the dilatation of the vessel wall, after BVS, and these include meticulous lesion preparation, gradual scaffold degradation, subsequent scaffold strut discontinuity, and consequential outward displacement of the scaffold, causing CAA formation. Further studies are warranted to quantify the true incidence and understand the underlying pathophysiological mechanism of CAA after BVS implantation, as well as with all types of stents, to address the challenges of managing such complications (56).

### Vasculitis

#### Kawasaki Disease

Kawasaki disease is the most common cause of CAAs in childhood and is the second most common cause in adults. KD is an acute inflammatory syndrome that may result in acute vasculitis of the coronary arteries and subsequent coronary artery dilatation and aneurysm formation; CAAs occur in around 10–15% of patients during the acute phase of the disease (64–66).

##### Inflammation in KD-Associated CAAs

Inflamed tissues in acute KD show infiltration of the arterial wall by mononuclear cells, lymphocytes, and macrophages; destruction of the internal elastic lamina; necrosis of the smooth muscle cells; myointimal proliferation; and subsequent dilations or aneurysms of the coronary arteries (65–68). The inflammatory cytokine TNF-alpha has been linked to the pathogenesis of CAA in KD patients, further supporting the role of inflammation in the development of CAAs. Rapid production of TNF-alpha in the peripheral immune system after disease induction in a murine model of KD has been shown to localize an immune response to the coronary arteries. In addition to that, the level of TNF-alpha remained elevated and was linked to the inflammatory process and breakdown of elastin that contributed to CAA formation. Moreover, pharmacological blockage of TNF-alpha effector mechanisms by using etanercept in these murine models was shown to eliminate these processes (69). An increased expression of soluble adhesion molecules in the sera of patients with coronary artery ectasias (CAEs) such as vascular cell adhesion molecule-1 and intercellular adhesion molecule-1 on the vascular endothelium, which play a role in the migration and adherence of immune cells (leukocytes) to the coronary arteries, further elucidates the inflammatory process’s role in the formation of CAA (70).

##### Genetic Contribution in KD-Associated CAAs

Several GWAS have contributed to the recent identification of various gene loci, which can help identify KD patients who are at an increased risk of developing CAAs, as well as recognize the extent of their vasculitis, to help initiate early diagnosis and robust treatment (67, 71–73). For example, Lin and colleagues investigated 64 patients with extremely large CAAs (diameter >8 mm) and compared them to 70 KD patients without CAAs; they revealed an association between the genetic variant rs2833195 in the intron of the TIAM1 gene, with the development and severity of CAA in KD. They postulated that the TIAM1 protein may play a role in chemokine-induced T cell migration and infiltration of lymphocytes into the vascular wall during the acute vasculitis stage of KD (71, 74). The genetic polymorphisms identified so far increase the risk of CAAs in KD patients by small amounts; therefore, to appreciate the extent of these and further undiscovered genetic susceptibilities, future studies are required to define the exact molecular mechanisms of CAA.

##### Matrix Metalloproteinase (MMP) Activity in KD-Associated CAAs

Histopathologic samples reveal the destruction of coronary artery walls with diffuse vasculitis, suggesting the involvement of MMPs in the formation of CAAs. MMPs are enzymes that can degrade the connective tissue proteins, leading to a weakened vascular wall (68, 75). MMP expression is regulated by transcription through a prostaglandin E2–cAMP pathway; inflammatory cytokines can stimulate this pathway resulting in an imbalance in the levels of MMPs. Tissue-specific inhibitors of MMPs (TIMP-1, TIMP-2, TIMP-3, and TIMP-4) are secreted by smooth muscle cells and regulate the activity of MMPs. Aneurysmal vessels have demonstrated an increased level of MMP-2, MMP-3, MMP-9, and MMP-12 and a reduced level of TIMPs; this proteolytic imbalance is thought to drive the degradation of vessel wall matrix and lead to CAAs (69, 70, 76). A study by Matsuyama et al. shows that the serum levels of MMP-3 and TIMP-1 are significantly higher in untreated KD patients than in a healthy control group of children (77). Moreover, some evidence highlights the role that genetic factors, particularly the variation in MMP-9 gene polymorphisms, play in the formation of aneurysms in KD (78). HLA-E and MMP-3 gene disruption, as well as insertion/deletion polymorphisms of the angiotensin-converting enzyme (ACE DD genotype), SRC-1 and GRIN3A genes are further possible genetic factors that have been linked to vessel wall weakening and the induction of coronary artery ectasias (72, 73, 79–82).

#### Other Vasculitic Disorders

Takayasu arteritis (TA) is a primary systemic vasculitis and involves the large cardiac vessels, mainly the aorta and its branches; less than 10% of patients with TA have CAAs (83). There are three main types of lesions seen in TA: stenosis or occlusion of the coronary ostia, diffuse or focal coronary arteritis, and coronary aneurysm (84). Systemic lupus erythematosus also commonly causes arteritis. Polyarteritis nodosa and rheumatoid arthritis are other vasculitic diseases that may lead CAA (4, 12, 22).

### Connective Tissue Disorders

Hereditary connective tissue disorders like Marfan syndrome and Ehlers–Danlos disease can also result in CAAs. Marfan syndrome is known to be associated with mutations in the fibrillin 1 (FBN1) gene (85). Fibrillin forms an essential element of the microfibrils that surround the elastin fibers, for maintaining vessel wall integrity (86). The FBN1 gene is homologous with the family of latent transforming growth factor-beta (TGF-b)-binding proteins, which maintains TGF-b in an inactive complex. Mutations of this gene may result in the overactivity of TGF-b and *via* mechanisms of cystic medial necrosis contribute to the formation of CAAs (30, 87). Mutations of the TGF-b receptor have been found to be associated with arterial aneurysms (88), and cystic medial necrosis has been commonly seen in aortic aneurysms in patients without Marfan syndrome (89), further supporting the overactivity of TGF-b as a driver of CAA formation.

Neurofibromatosis (NF) is a congenital hereditary disease with generalized neuroectodermal and mesodermal dysplasia that affects the skin and multiple systems such as the nervous, skeletal and vascular system. There have been a few rare documented NF cases who suffered a myocardial infarction because of thromboembolism (90–92).

### Infections and Drugs Use

Bacterial, mycobacterial, fungal, syphilitic, Lyme, septic emboli, mycotic aneurysm, and HIV infections are also different CAA etiologies. The direct invasion of pathogens into the vessel wall or immune complex deposition are also known drivers of the CAAs in mycotic lesions (93). Some investigators have reported that mycotic CAAs after DES implantation are also possible (94). Use of drugs such as cocaine, protease inhibitors, amphetamines is additionally responsible for CAAs (22, 95). The potential mechanism of CAA formation among cocaine users may be from severe hypertension episodes and the direct endothelial damage caused by vasoconstriction (96).

### Other Causes of CAAs

Congenital CAA is another etiology of CAAs, which accounts for 20–30% of all coronary aneurysms; however, the formation of congenital CAAs remains poorly understood (97).

Fibromuscular dysplasia (FMD), a non-atherosclerotic and non-inflammatory vascular disease, commonly associated with lesions of the internal carotid and renal arteries, has also been described in the coronary arteries (98). For the first time, a CAA was reported as a complication of FMD of the intimal, medial, adventitial, and periarterial layers (99). This phenomenon could further the understanding of the underlying mechanisms of congenital CAAs.

## Clinical Presentation

**Figure 1:**
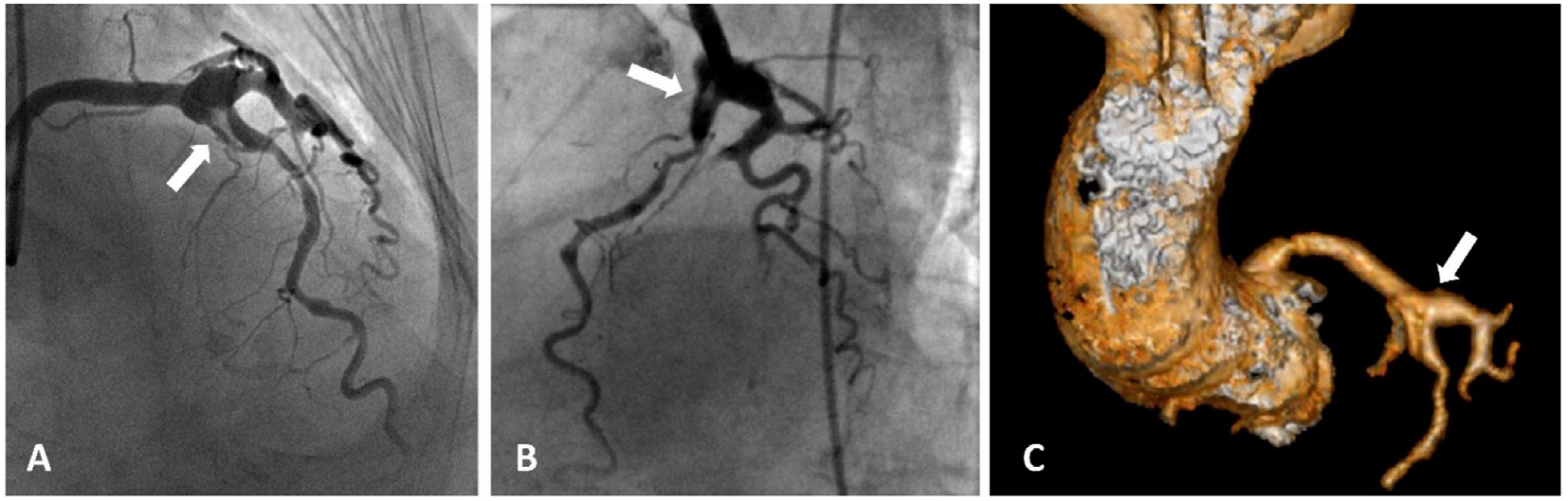
**Acute coronary syndrome and coronary artery aneurysms**. A patient presented with acute coronary aneurysm. **(A,B)** Coronary angiography revealed an aneurysmal dilatation of the ostia of the left anterior descending (LAD) artery and left circumflex artery, with a thrombus image (arrow heads) in the LAD artery along with a significant stenosis in the proximal part. Control angiogram failed to show any reduction in the thrombus after 48 h of tirofiban infusion, and the patient was referred to the surgery. **(C)** Aneurysmal dilatation can also be seen in computed tomography images.

**Figure 2:**
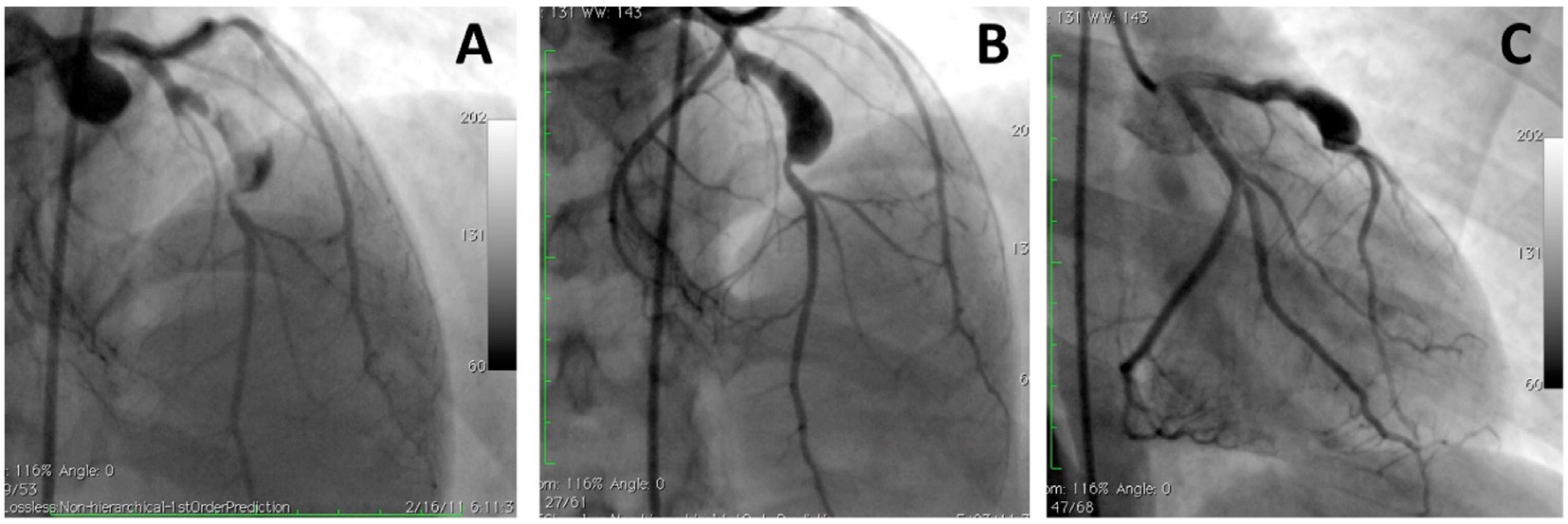
**A large aneurysm in left anterior descending artery in a 17-year-old patient with neurofibromatosis causing acute coronary syndrome**. Coronary angiograms with **(A)** and without **(B,C)** thrombus image in the aneurysm after anticoagulation. However, this patient was referred to surgery to prevent future complications [with permission from the report by Evrengul et al. (92)].

In most cases, CAAs are asymptomatic, and when symptomatic, the clinical manifestations vary and are typically reliant on the underlying cause. Most often, the clinical manifestations of CAAs are like those seen in coronary artery disease (3, 8, 100). A frequent finding is the presence of thrombi within the aneurysm. The slow flow of blood on the irregular internal surface of the aneurysm wall predisposes the formation of thrombi with subsequent embolization, resulting in angina pectoris (Figures 1 and 2), dyspnea, myocardial ischemia, and/or infarction and sudden death (26, 100–102). Angina can also be related to the associated atherosclerosis or the sluggish flow caused by the aneurysm itself. In some instances, a murmur can be auscultated (16, 103, 104). CAAs have also been reported in association with an abdominal aortic aneurysm or hypertension. Rupture of the aneurysm is a rare occurrence, nonetheless, a potentially catastrophic complication of a CAA. Patients with giant CAAs can present in a variety of ways, such as with superior vena cava syndrome or with a mediastinal mass, which can often be misdiagnosed as a cardiac tumor (16, 21, 104, 105). Large aneurysms are a diagnostic challenge (105); the differential diagnoses for these include cysts and other masses (100). The variation in the presentation of CAAs highlights the importance of utilizing a variety of diagnostic approaches.

## Diagnosis

Coronary artery aneurysms are diagnosed using various imaging techniques, and the ideal methods should evaluate the distribution, maximal diameter, presence or absence of intraluminal thrombi, and the number and extension of aneurysms and associated complications, such as a myocardial infarction. Since most of the patients with CAAs remain asymptomatic, aneurysms are mostly incidentally found during diagnostic coronary angiography (4). Although coronary angiography, incorporated with intravascular ultrasound (IVUS), remains the conventional method for the assessment of the coronary anatomy and identification of CAAs, the limitations of this technique have highlighted the use of other diagnostic approaches in the context of CAAs. Furthermore, CAAs may also be detected by non-invasive tools including echocardiography, CT, and MR imaging (60, 102).

### Coronary Angiography

Coronary angiography provides important information about the size, shape, location, and frequency of aneurysms, as well as the degree of coronary artery atherosclerosis (2, 106, 107). As well as evaluating any coronary artery stenoses, coronary angiography is also able to detect thrombotic occlusions and determine the extent of collateral artery formation. Prior to referring a patient for invasive angiography, it is important to consider the benefits and risks of the procedure, particularly in younger patients with KD. While coronary angiography may not provide further information in patients with mild ectasia or small fusiform aneurysms, it can play a larger role in more complex coronary lesions. Also, conventional angiography is limited to a “luminogram” and does not reflect information about the vessel wall, and this could lead to the underestimation of the actual size of aneurysm or even overlook a CAA that may be occluded by a large thrombus or a plaque. Coronary angiography is also limited in its ability to differentiate between a true aneurysm and a pseudoaneurysm (108–110).

### Intravascular Ultrasound

Intravascular ultrasound can be incorporated in the invasive angiographic evaluation of CAAs, and treatment of CAAs can be initiated during the same procedure (20). IVUS has become the “gold standard” for providing critical anatomical information to address the diagnostic limitations of evaluating coronary aneurysms during angiography (108). Intravascular ultrasonography provides transmural images of the coronary arteries, allowing for information on the arterial wall structure and luminal composition to be derived (109, 110). This is important for the differentiation between the different types of aneurysms, which vary in prognosis. Pseudoaneurysms are characterized by a loss in the vessel wall integrity, resulting in a highly rupture-prone adventitia and perivascular tissue; most perforation-related enlargements are pseudoaneurysms. On the other hand, most postcoronary intervention aneurysms are true aneurysms that follow a healed dissection (109). One study used intravascular ultrasound to assess coronary artery aneuryms diagnosed by angiography in 77 patients. Only a third of angiographically confirmed CAAs were true or pseudoaneurysms, 27 and 4% respectively. IVUS could reveal that most of the remainder of these aneurysms, 16%, in fact, had the morphology of complex plaques or were just normal segments adjacent to stenotic regions, 53% (110). Moreover, IVUS is also useful for clarifying the relationship of a coronary aneurysm with a previously implanted stent, and this is important given the recent association of CAAs with percutaneous coronary interventions. Aneurysms can develop outside the stent struts when the stent has been implanted for the treatment of a coronary dissection (111). IVUS can also be used as an adjunct during cardiac catheterization to detect structural or functional changes in the coronary artery wall (110). IVUS on the angiographically documented regressed CAAs demonstrates unusual thickening of the intima-media complex as well as a reduced vasoactive response to vasodilators (112–114). The long-term clinical implications of these anatomical and functional changes are currently unknown and may be underline fundamental features in the natural history of CAAs (111). IVUS can also be used during the treatment of CAAs; it proves to be an invaluable tool in guiding the adequate coverage of aneurysms (108).

### CT Angiography

Most recently, multidetector-row computed tomography (MDCT) technology has led to an increased interest for the use of non-invasive coronary angiography. MDCT has been utilized in the context of CAAs, despite its ill-defined role in the management of patients with cardiovascular symptoms (115). In fact, the advent of MDCT has increased the incidental finding of coronary aneurysms and ectasia, which were previously a rare finding on coronary angiography (3). CT data acquisitions can be reconstructed to provide information regarding the nature of the dilatation in the coronary vessel, such as the maximum diameter, shape, morphology, and presence of any concomitant stenosis, plaque composition, and its location in relation to the surrounding vasculature (4). The limitations of coronary angiography in the detection of coronary aneurysms (i.e., underestimation of CAA size in the presence of intraluminal thrombi) have fueled investigators to assess the prospective role of CTCA in the diagnosis of CAA. Recently, Forte et al. retrospectively analyzed 390 consecutive CTCAs performed from 2007 to 2015 for suspected CAD or follow-up after myocardial ischemia; they divided patients into two groups, those with and those without CAAs. CTCA could identify aneurysmal thrombus in five patients (55.5%), and a significant difference in the diameter of the coronary vessels was found between the two groups of patients (116).

CTCA provides a fast three-dimensional evaluation that helps to provide an easy understanding of complex anatomic structures, such as those seen in ectasia associated with coronary fistulas, as well as allow for the analysis of the lumen composition and the vessel wall. While coronary angiography allows for both diagnosis and treatment of CAAs, CTCA could be used for the follow-up of patients with suspected or treated CAAs.

Nonetheless, this method requires the exposure to radiation and the use of iodinated contrast media; such issues should be considered when evaluating young patients who require follow-up investigations, as well as patients with renal failure. To address this, prospective electrocardiographically gated scans and dose modulation techniques have been devised to reduce the radiation (117, 118). Nonetheless, there are limited data published from large series studies regarding the coronary CT angiography in the diagnosis of CAAs (4). Further studies are required to compare the sensitivity and specificity of CTCA in the detection of CAAs compared to that of the current “gold standard” to help postulate its prospective role in the diagnosis of CAAs.

### Echocardiography

Other non-invasive methods, useful for the diagnosis of CAAs, include transthoracic echocardiography and transesophageal echocardiography (107). The non-invasive nature of echocardiography, as well as its high sensitivity and specificity for the detection of abnormalities in the proximal LMCA and RCA, makes it an ideal imaging modality for the assessment of the cardiac sequel of KD in children. Echocardiography allows for the quantitative assessment of the internal vessel diameters, providing information on the location of the aneurysms and the presence or absence of intraluminal thrombi. Depending on the relation of the axial and lateral diameters of the coronary vessels to each other, one can identify the nature of the aneurysm. Echocardiography is also a useful non-invasive method to guide the long-term follow-up of these patients into adulthood, alongside coronary angiography; it has also been suggested that the decision to perform angiography may be guided by echocardiographic imaging of coronary arteries (111).

### Coronary MR Angiography (MRA)

As previously mentioned, echocardiographic evaluation becomes increasingly limited as children grow up (107); here, coronary MRA is a useful alternative. MRA may also be a useful substitute in patients for whom the use of other non-invasive modalities may be contraindicated. MRA may describe CAAs in proximal coronary artery segments and feedback information regarding flow rate and character (119, 120). A recent series on KD patients demonstrated the ability of MRA in effectively diagnosing all CAAs, occlusions, and stenoses, which were found on coronary angiography (121). Current literature supports the ability of MRA to investigate large proximal segments of the coronary vasculature; however, the resolution of MRA is reduced when it comes to imaging the smaller distal segments. Therefore, additional studies in patients with KD and CAAs are needed to establish the reliability of MRA in the diagnosis of CAAs and stenoses in distal arterial segments (122).

## Treatment

There is no consensus on the optimal management of CAAs, and the treatment options for CAAs consist of surgical, percutaneous, and medical approaches. Since the natural history and prognosis are related to multiple factors, the decisions around treatment should be tailored to each patient and should consider many aspects such as the clinical presentation, etiology, aneurysm size, location and its expansion by time, association with infections, and the presence and extent of any coexisting atherosclerosis (12, 19, 76, 123, 124).

### Medical Treatment

Patients with atherosclerosis should receive guideline-directed medical therapy to modify their coronary artery disease risk factors. The possible link between the inflammatory cytokines and MMPs with CAAs may also indicate a role for statins and inhibition of the renin–angiotensin system (30); however, there are no studies, to date, supporting these hypotheses. If thrombosis and/or embolism is a concern, the long-term use of antiplatelet and anticoagulant therapy should be considered (25).

Intravenous immunoglobulin (IVIG) therapy is used in patients with Kawasaki disease for the treatment of coronary artery aneurysms. The early initiation of treatment and a smaller sized coronary artery aneurysm at diagnosis, have both been associated with a higher rate of CAA regression as well as a lower incidence of major adverse-coronary events (125–127). Although the use of IVIG in KD patients is beyond the scope of this review, it is important to mention these recent findings that have highlighted the prognostic benefits of the early recognition and treatment of KD-associated CAAs and emphasized the need for further studies to comprehend the identifiable risk factors and predispositions (such as the genetic susceptibility loci previously discussed) to developing CAAs.

### Percutaneous Intervention

**Figure 3:**
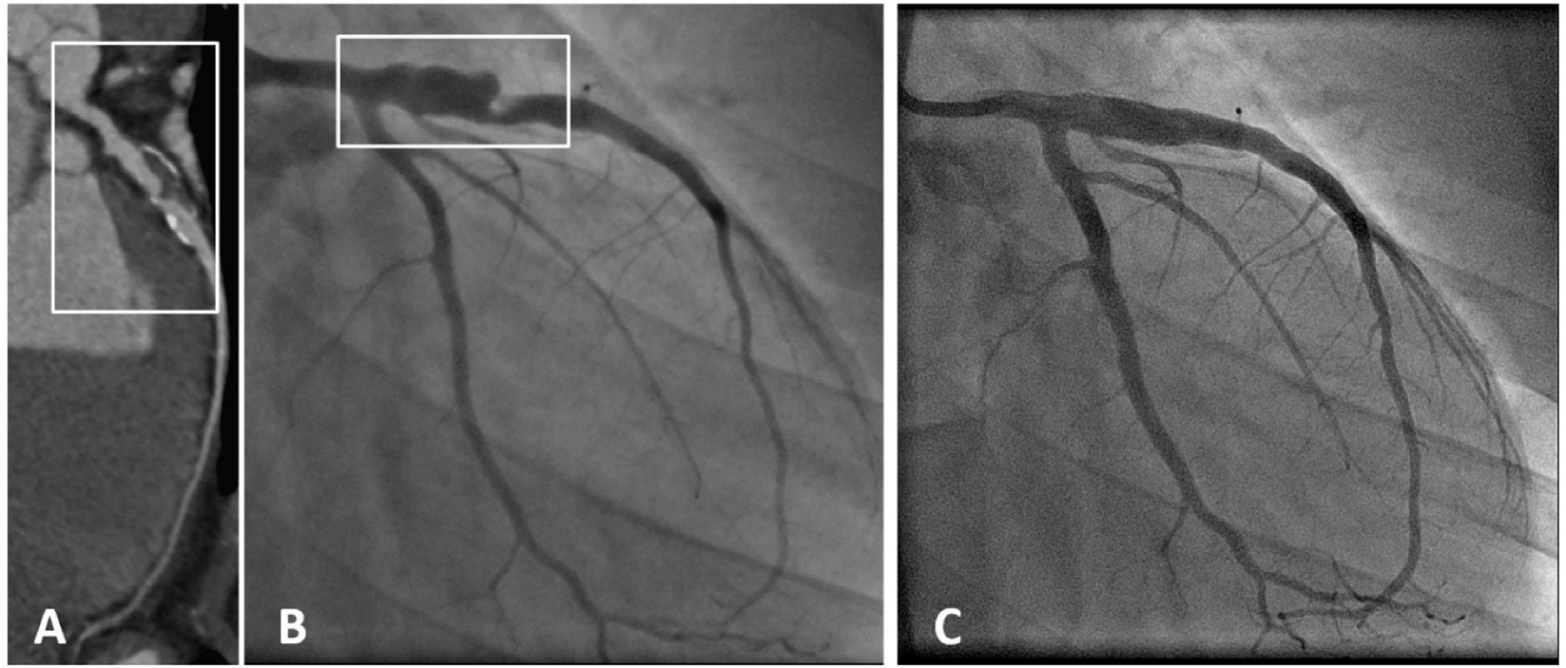
**A patient with coronary aneurysm due to Kawasaki disease**. **(A)** Computed tomography angiography showed a large aneurysm (maximum diameter 9 mm, extending for more than 35 mm) of the proximal left anterior descending (LAD) artery with calcific degeneration, large filling defect with thrombus and severe lumen narrowing. **(B)** Coronary angiography confirmed a 70% stenosis of the LAD artery with an aneurysm immediately distal to the ostium (maximum diameter 9.2 mm × 7.8 mm). **(C)** Final angiography demonstrating exclusion of the aneurysm using a covered stent [with permission from the report by Di Mario et al. (133)].

In some cases, a decision can be made to exclude the aneurysm to prevent future complications. During decision making, it is important to weigh both the immediate and the long-term risks that surround percutaneous interventions or surgery versus the complications that can occur on prolonged antithrombotic medication, such as bleeding. In the instance where percutaneous exclusion has been decided upon, covered stents are recommended, in those with the suitable anatomy (Figure 3) (128, 129). However, concerns around the use of covered stents still exist and should also be considered, and these include a reduced deliverability, the risk of restenosis and thrombosis, and occlusion of side branches (130). In a previous case series of seven CAA patients, Briguori et al. treated them with (PTFE)-covered stents; at 35 ± 8 months of clinical follow-up, six patients were found to be symptom free. During the angiographic follow-up at 10 ± 6 months, restenosis occurred in only one patient (131). In an analysis of the previous cases in 2005, authors reported that only 5 of the 24 patients who received stents were found to have restenosis on follow-up angiography; these patients had larger aneurysms (>10 mm in diameter) (132). These results were compared with a preceding study that reviewed patients undergoing surgical treatment for CAA, and they recommended the use of covered stents for small-sized CAAs and surgery for large aneurysms (greater than 10 mm); however, this study was relatively small, lacked controls, and had heterogeneous data.

Another percutaneous option in the management of wide-necked aneurysms is coil insertion (134, 135); however, experience is needed to perform these interventions. Also, coil herniation can lead to the occlusion of the parent vessel; therefore, stent-assisted techniques are preferred. Another pitfall is the risk of aneurysm rupture during the manipulation of the microcatheter, coils, or wires (134).

### Surgery

**Figure 4:**
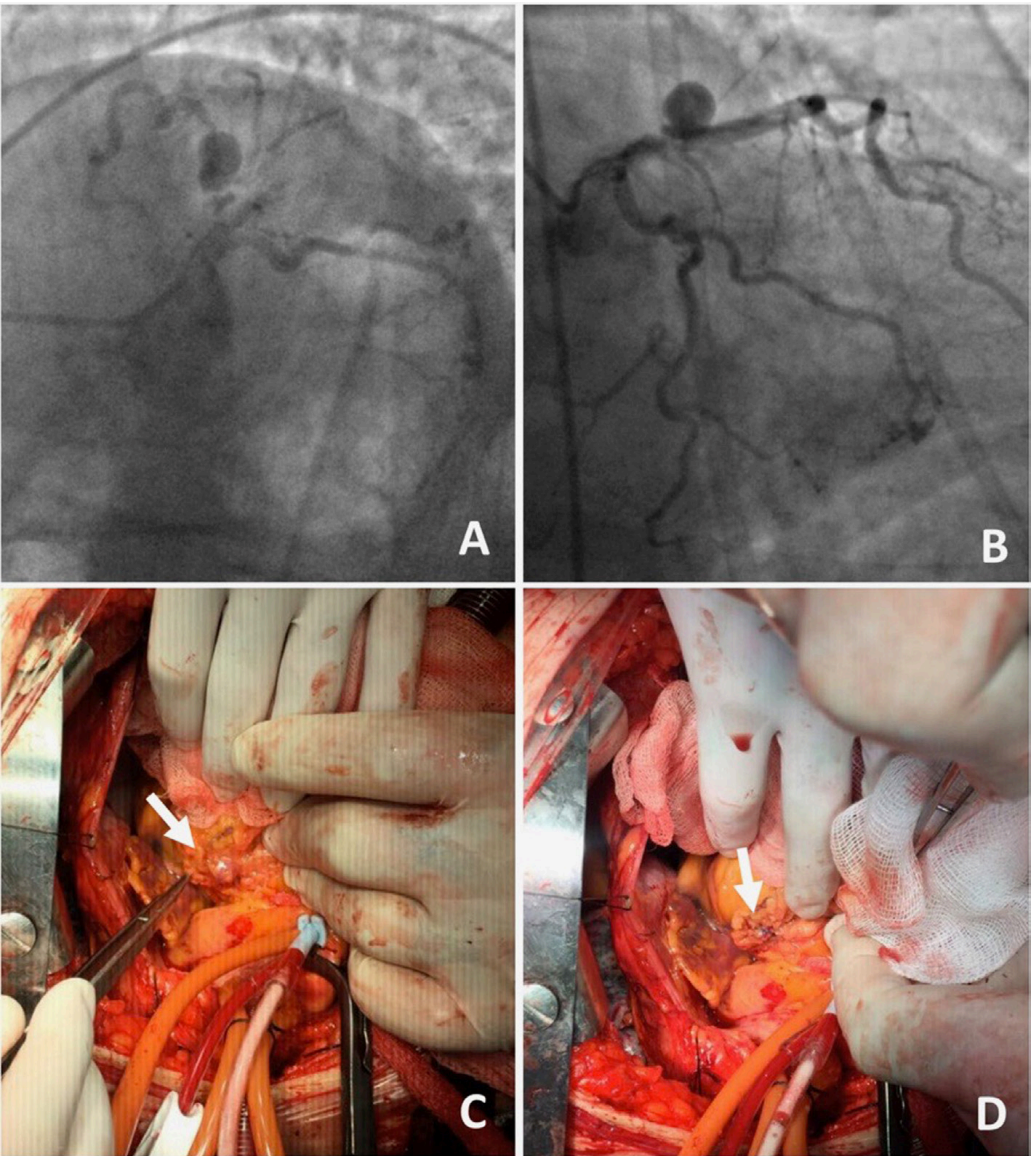
**A 70-year-old male presented with angina on exertion**. Three years prior to this presentation, they had an abdominal aortic aneurysm repair.**(A,B)** Coronary angiogram revealed coronary artery disease and an aneurysm in the left anterior descending artery. The patient was referred to surgery, because of the risk of occlusion of the significant side branches, if a stent was to be used. **(C,D)** Coronary aneurysm was surgically excluded with a linear repair (images courtesy of Yalin Tolga Yaylali and Bilgin Emrecan).

Finally, surgery is an alternative for patients whom cannot be treated percutaneously. It is also indicated in patients with obstructive coronary artery disease or in individuals with large saccular aneurysms at a high risk of rupturing (107). Several procedures can be performed during surgery, and these include resection of the aneurysm, proximal and/or distal ligation (Figure 4), aneurysmal thrombectomy, and aneurysmectomy with or without bypass grafting (128).

## Conclusion

In retrospect, while our understanding of CAAs has developed over the last few years, a great deal remains unknown. CAAs are rare; nonetheless, it can result in fatal outcomes in patients. CAAs are most commonly associated with coronary artery disease, and there is a growing concern regarding the development of CAAs following percutaneous coronary interventions. Several studies have postulated the underlying pathophysiology of CAA; however, the specific mechanisms that drive the formation of CAAs remain unclear. Despite the multifactorial nature of CAA, the recent discovery of genetic polymorphisms that may increase the risk of CAA in patients such as those with KD, in addition to the prognostic benefits of the early diagnosis and treatment of CAAs, emphasizes the need for further studies to consolidate any identifiable risk factors to help target those at risk of CAAs and subsequent complications. With more widespread use of angiography, high-resolution CT, and MRI, the diagnosis of CAA is expected to increase; therefore, robust evidence-based management strategies are vital. The management of CAAs remains a clinical challenge and should be tailored to each patient based on a comprehensive clinical evaluation that encompasses the patient’s cardiovascular risk factors, comorbidities, and the nature and anatomy of the CAA to enable a patient-specific treatment plan. Certainly, as the understanding of the pathophysiology and etiology of CAAs develops, the treatment and prognosis of CAAs will improve.

## Author Contributions

SS: literature search, writing etiology, clinical presentation, and diagnosis. OT: literature search and writing treatment. ÖT: literature search, writing introduction classification, and epidemiology. IK: literature search and reviewed the paper. OG: critical review of paper.

## Conflict of Interest Statement

The authors declare that the research was conducted in the absence of any commercial or financial relationships that could be construed as a potential conflict of interest.
